# Inverse correlation between Interleukin-34 and gastric cancer, a potential biomarker for prognosis

**DOI:** 10.1186/s13578-020-00454-8

**Published:** 2020-08-04

**Authors:** Qinghua Liu, Ying Zhang, Jiwei Zhang, Kun Tao, Brett D. Hambly, Shisan Bao

**Affiliations:** 1 Department of Pathology, Xuzhou Medical University, Xuzhou, 221000 China; 2 Discipline of Pathology, Bosch Institute and School of Medical Sciences, Charles Perkins Center D17, Sydney Medical School, The University of Sydney, Sydney, NSW 2006 Australia; 3 Department of Surgery, Songjiang Hospital, Shanghai Jiaotong University School of Medicine, Shanghai, 201600 China; 4 Tongren Hospital, Shanghai Jiaotong University, School of Medicine, Shanghai, 200336 China

## Abstract

**Background:**

Gastric cancer (GC) is a malignancy with high morbidity/mortality, partly due to a lack of reliable biomarkers for early diagnosis. It is important to develop reliable biomarker(s) with specificity, sensitivity and convenience for early diagnosis. The role of tumour-associated macrophages (TAMs) and survival of GC patients are controversial. Macrophage colony stimulating factor (MCSF) regulates monocytes/macrophages. Elevated MCSF is correlated with invasion, metastasis and poor survival of tumour patients. IL-34, a ligand of the M-CSF receptor, acts as a “twin” to M-CSF, demonstrating overlapping and complimentary actions. IL-34 involvement in tumours is controversial, possibly due to the levels of M-CSF receptors. While the IL-34/M-CSF/M-CSFR axis is very important for regulating macrophage differentiation, the specific interplay between these cytokines, macrophages and tumour development is unclear.

**Methods:**

A multi-factorial evaluation could provide more objective utility, particularly for either prediction and/or prognosis of gastric cancer. Precision medicine requires molecular diagnosis to determine the specifically mutant function of tumours, and is becoming popular in the treatment of malignancy. Therefore, elucidating specific molecular signalling pathways in specific cancers facilitates the success of a precision medicine approach. Gastric cancer tissue arrays were generated from stomach samples with TNM stage, invasion depth and the demography of these patients (n = 185). Using immunohistochemistry/histopathology, M-CSF, IL-34 and macrophages were determined.

**Results:**

We found that IL-34 may serve as a predictive biomarker, but not as an independent, prognostic factor in GC; M-CSF inversely correlated with survival of GC in TNM III–IV subtypes. Increased CD68^+^ TAMs were a good prognostic factor in some cases and could be used as an independent prognostic factor in male T3 stage GC.

**Conclusion:**

Our data support the potency of IL-34, M-CSF, TAMs and the combination of IL-34/TAMs as novel biological markers for GC, and may provide new insight for both diagnosis and cellular therapy of GC.

## Background

Gastric cancer (GC) is an important disease with high morbidity and mortality. Due to a lack of relatively convenient and reliable biomarkers, large numbers of GC are diagnosed at an advanced stage, with poor prognosis [1]. It is fundamentally important to develop reliable biomarker(s) with enough specificity, sensitivity and convenience for early diagnosis. Whilst cell-mediated immunity may exhibit anti-tumour activity, epidemiological, preclinical and clinical studies demonstrate that chronic inflammation plays a vital role in the initiation and/or development of gastric cancer [2]. Chronic inflammation mediates tumourigenesis, including cellular survival, proliferation, migration, angiogenesis and metastasis via cytokine mediated signalling pathways.

The inflammatory microenvironment surrounding a tumour is a complex ecology of immune cells interconnected with tumour cells. Among the leucocytes present at the tumour site, macrophages are abundantly present at all stages of tumour progression [3]. Tumour-associated macrophages (TAMs) are correlated with poor survival of GC patients, as TAMs promote invasion and metastasis through enhancing angiogenesis [4]. However, others have reported a positive correlation between TAMs and 5 year survival rate of GC patients [5]. Multiple factors may contribute to this discrepancy, including tumour type and stage [6].

Macrophage colony stimulating factor (M-CSF) is a growth factor important in the regulation of differentiation, proliferation and survival of haematopoietic cell lineages [7]. Circulating M-CSF is increased in many tumours (e.g. breast, prostate and pancreatic cancers) and is positively correlated with invasion, metastasis and poor survival of tumour patients [8–10]. By contrast, monocytes/macrophages are able to kill cancerous cells by paraptosis, driven by over-expression of membrane M-CSF [11].

IL-34 was first identified by Lin et al. in 2008, as a protein that is able to bind to CD14^+^ monocytes in peripheral blood mononuclear cells. IL-34 stimulates the differentiation of monocytes into macrophages via the CSF-1 receptor [12]. Subsequently, IL-34, including mRNA and protein, can be detected in various tissues secreted by fibroblast-like cells. The order of the level of production in the tissues is: spleen, heart, brain, thymus, lung, kidney, liver, small intestine, colon, testes, ovary and prostate [13]. Using an IL-34 reporter gene, for IL-34 a high level of expression has been detected in the skin and in the brain compared to other non-lymphoid and lymphoid tissues [14].

IL-34 is also a ligand of the M-CSF receptor, and acts as a “twin” to the M-CSF cytokine, demonstrating overlapping and complimentary actions [15]. IL-34 acts similarly to MCSF in promoting osteoclastic differentiation of giant cell tumours [16], but IL-34 also displays singular function during brain development [17]. Furthermore, the role of IL-34 in tumours is controversial particularly in the development, metastasis and prognosis of cancers, although the response to MCSF is tumour-type dependant, possibly due to the levels of M-CSF receptors [18].

Studies have long sought specific biological markers that could characterize GC [19]. However, no existing marker(s) have proven to be sufficiently specific to GC. While the IL-34/M-CSF/MCSFR axis is very important for regulating macrophage differentiation [20], the specific interplay between these cytokines, macrophages and the development of tumours is unclear. Accordingly, a multi-factor evaluation could provide more objective utility, particularly for either prediction and/or prognosis of gastric cancer, compared to studies in the current literature.

Conventional chemotherapy kills cancers non-specifically, based on the high rate of cancer cell division. Precision medicine is becoming popular in the treatment of malignancy, which tailors intervention to the individual patient with customization of medical decisions and healthcare [21]. However, the success of a precision medicine approach replies on the identification of highly specific targets in each specific tumour. Therefore, elucidating specific molecular signalling pathways in specific cancers is necessary to facilitate the success of a precision medicine approach.

In our current study, we determined the production of M-CSF and IL-34, and the number of infiltrating CD68^+^-TAMs in GC. The relationship between M-CSF, IL-34 and CD68^+^-TAMs infiltration in GC was explored with a view to elucidate potential molecular targets.

## Results

### IL-34, M-CSF and CD68^+^-TAMs in GC

**Fig. 1:**
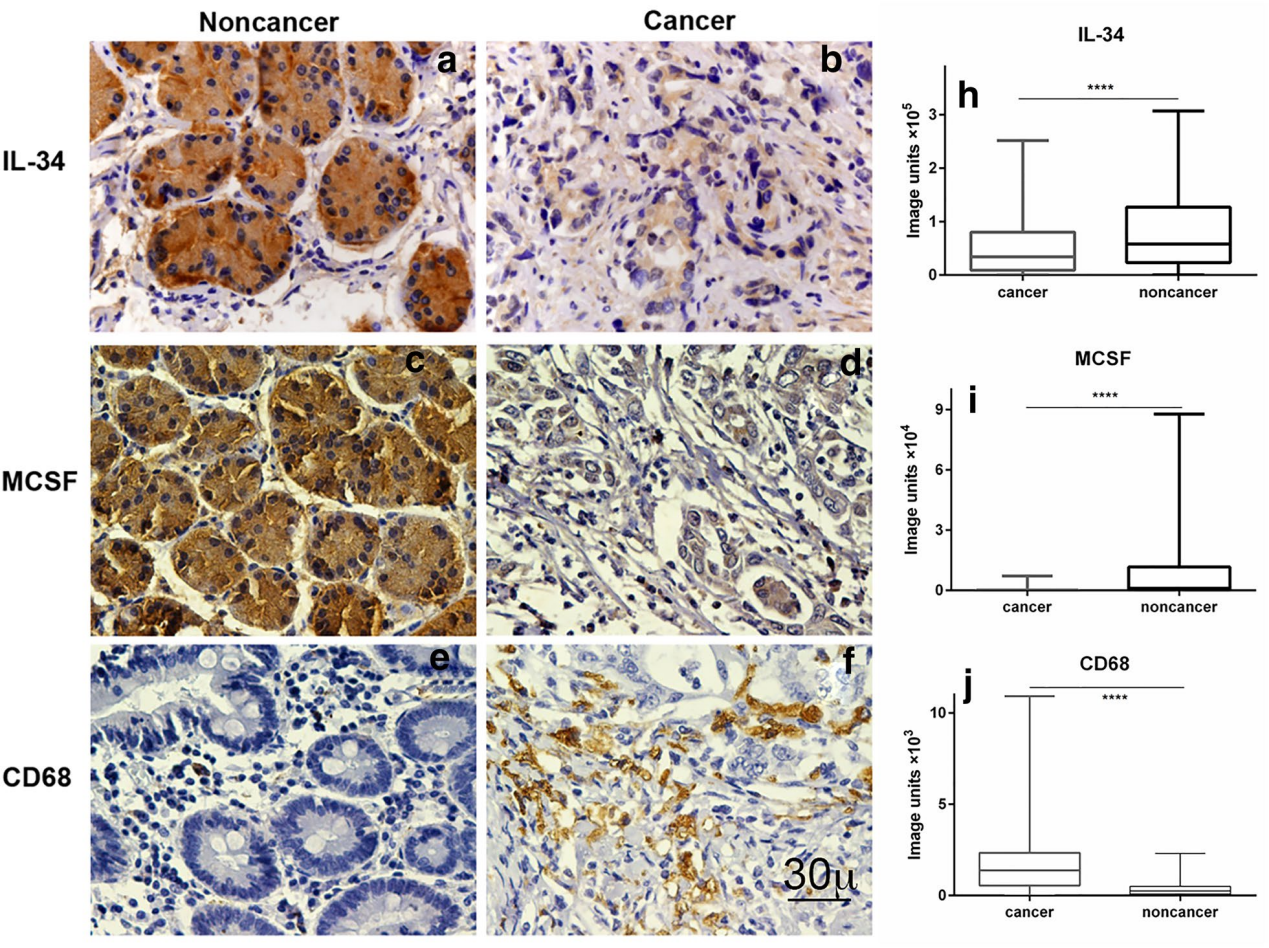
Representative images of IL-34, MCSF and CD68^+^ TAMs immunohistochemistry staining and their densities in noncancerous and GC tissues. Positive IL-34, MCSF and CD68^+^ TAMs were stained in brown mainly expressed in the cytoplasm of gastric cancer and noncancerous tissues. The densities of IL-34 and MCSF were decreased in GC compared to tumour adjacent gastric tissues, whereas the density of CD68 was increased. Magnification, ×600

The expression levels of IL-34, M-CSF and CD68^+^-TAMs in GC were investigated. Following immunohistochemical staining (Fig. 1), the densities of IL-34, M-CSF and the number of CD68^+^-TAMs were determined and are presented as box plots, including medians and 25th and 75th percentiles. IL-34 and M-CSF were decreased > 40% and > 95%, respectively, in GC compared to tumour adjacent gastric tissues (p < 0.05), whereas CD68^+^-TAMs were increased 5.5 fold (p < 0.05) (Fig. 1).

### Correlation between IL-34, M-CSF and CD68^+^-TAMs in GC and clinicopathological parameters

**Fig. 2:**
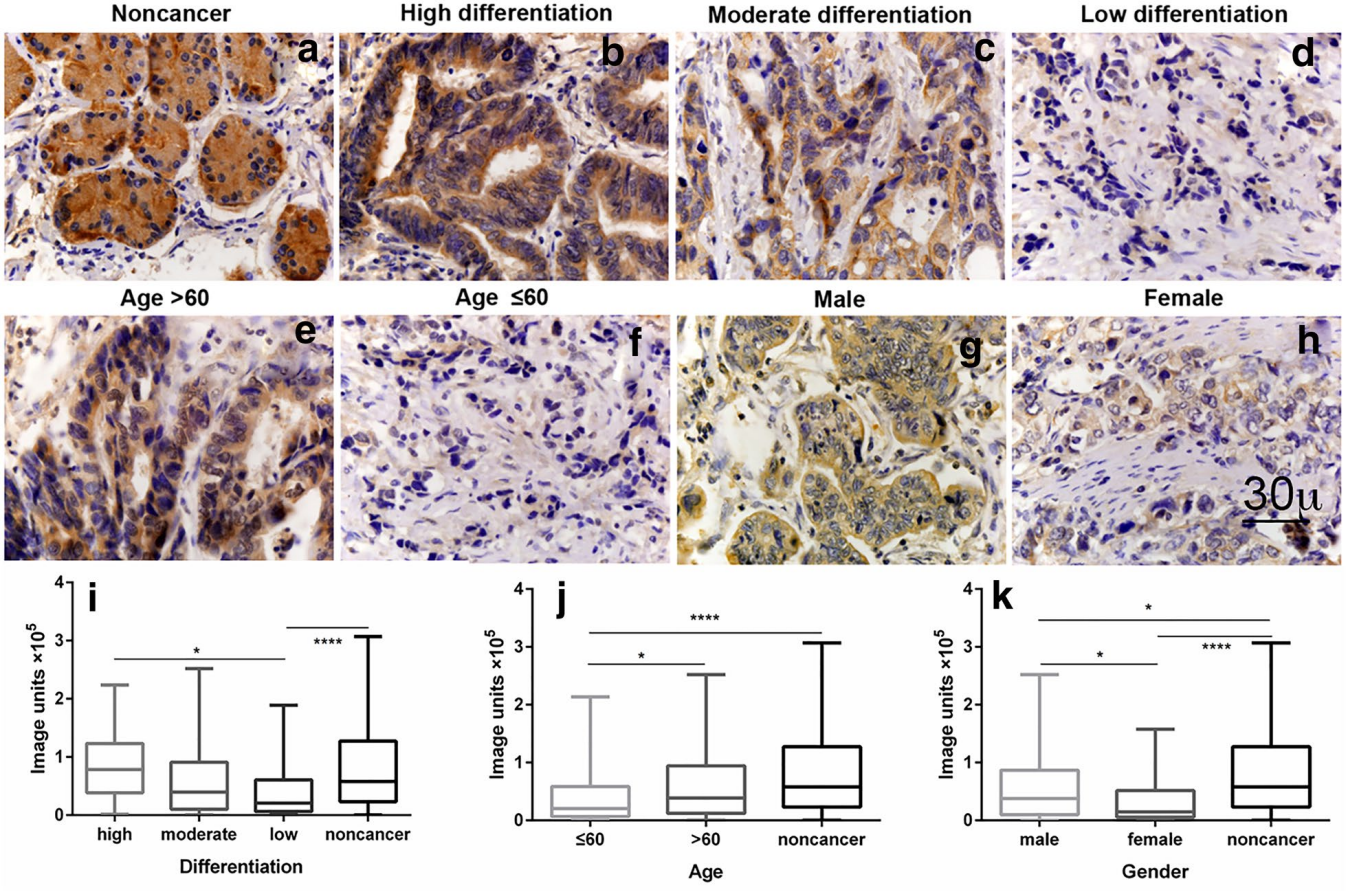
Correlation of IL-34 expression with age, gender and differentiation subtypes. IL-34 decreased in the groups of GC patients aged less than or equal to 60 years old, female and low differentiation subtypes of GC

The median values obtained for IL-34, M-CSF and CD68^+^-TAMs expressions were found to significantly differ between subgroups defined by a range of clinicopathological parameters (Table 1, Fig. 2 and Additional file 1: Figure S1, Additional file 2: Figure S2). The median expression of IL-34 differed significantly with age, gender and tumour differentiation of GC patients (Fig. 2). There was less IL-34 (decreased 46.70%) in the group of patients aged ≤ 60 years compared to patients aged > 60 (*p *< 0.05). Lower IL-34 (decreased 61%) was also observed in female compared to male GC patients (*p *< 0.05). In addition, there was a significant correlation between IL-34 and differentiation of GC (low differentiation group of GC had 74% decreased IL-34 compared to the high differentiation group) (*p *< 0.05), suggesting that IL-34 correlates with the state of differentiation of GC. There was no correlation between IL-34 and other parameters, such as tumour size, lymph node metastasis, tumour invasion depth and TNM stage of GC. Additionally, there was no correlation between clinical parameters and M-CSF or CD68^+^-TAMs (Additional file 1: Figure S1, Additional file 2: Figure S2).

**Table 1:** Correlations between IL-34, MCSF and CD68^+^ TAMs and clinical pathological features in patients with GC (n = 180)

Characteristics	Patients	IL-34		CD68		MCSF	
		Median	*p*	Median	*p*	Median	*p*
All cancer	180	33,827	< 0.001	1366	< 0.001	36.61	< 0.001
Noncancer (non)	159	57,921		246.0		1007	
Gender							
Male	140	37,935	0.030	1325	> 0.999	38.02	> 0.999
Female	40	14,847		1535		27.25	
Age							
≤ 60	79	20,658	0.048	1066	0.083	25.53	0.742
> 60	101	38,759		1702		48.79	
Tumour size (diameter)							
< 5 cm	87	40,818	0.358	1418	> 0.999	45.35	> 0.999
≥ 5 cm	93	24,419		1321		32.54	
Lymph node metastasis							
No	75	43,296	> 0.999	1715	0.169	48.20	> 0.999
Yes	105	30,495		1185		28.30	
Differentiation							
High	14	78,613	H/L: 0.043 H/M: 0.499 M/L: 0.495	1250	All > 0.999	223.8	H/L: 0.051 H/M: 0.471 M/L: 0.791
Moderate	78	39,376		1757		40.12	
Low	88	20,788		1186		26.00	
Tumour invasion depth							
T1	4	87,693	All > 0.999	1762	All > 0.999	20.82	All > 0.999
T2	27	51,850		1644		26.86	
T3	75	27,518		1593		40.12	
T4	74	32,160		1101		39.80	
TNM							
I	12	22,371	I/II, I/III I/IV, II/III > 0.999 II/IV: 0.278 III/IV: 0.643	1644	I/II, I/III I/IV, II/IV III/IV > 0.999 II/III: 0.636	27.25	I/II, I/III II/III > 0.999 I/IV: 0.942 II/IV: 0.277 III/IV: 0.183
II	70	42,580		1564		48.79	
III	92	32,051		1147		42.15	
IV	6	6544		936.6		3.390

### Correlation of decreased IL-34, M-CSF but increased CD68^+^-TAMs with overall survival of GC patients

**Fig. 3:**
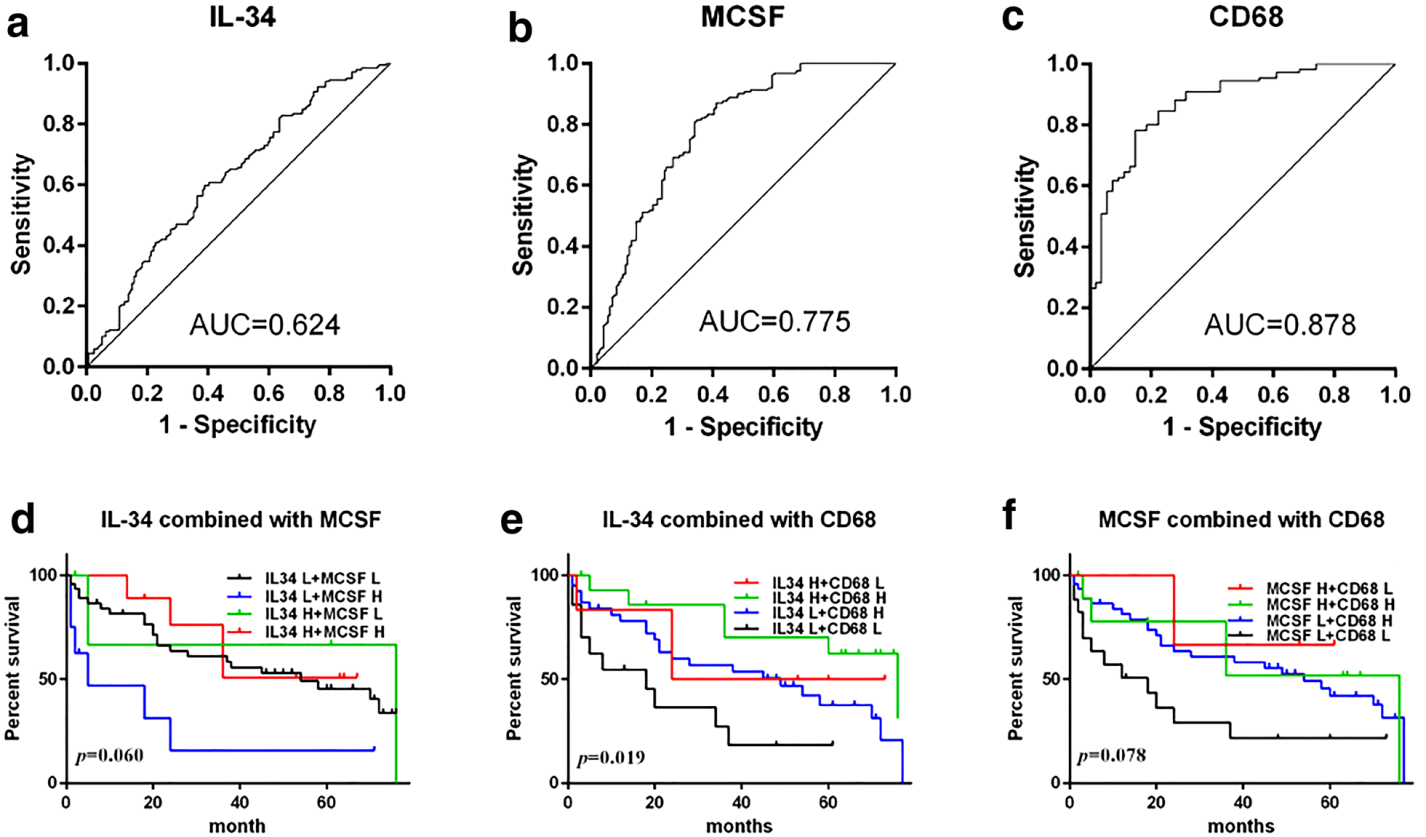
ROC and combination analysis of IL-34, MCSF and CD68^+^ TAMs for prognosis of GC. ROC curves analysis displaying the diagnostic of GC by expression levels of IL-34, MCSF and CD68. Area under the curve, IL-34: 48,550, AUC = 0.624; MCSF: 233.7, AUC = 0.775; CD68: 768.7, AUC = 0.878. Kaplan–Meier survival analysis of combination of IL-34, MCSF and CD68^+^ TAMs for prognosis of GC, the combination of IL-34 and CD68^+^ TAMs had relationship with the prognosis of GC patients

To evaluate whether decreased IL-34, M-CSF and increased CD68^+^-TAMs correlated with survival of GC patients, low and high cut-off points for IL-34, M-CSF and CD68 were defined by ROC curve analysis (Fig. 3).

The area under the curve (AUC) derived from the ROC curves showed that CD68^+^-TAM was the most sensitive marker for prognosis (AUC = 0.878), demonstrating a moderate to high accuracy, while IL-34 (AUC = 0.624) and M-CSF (AUC = 0.775) demonstrated only moderate accuracy [22].

Kaplan–Meier analysis was further applied to compare overall survival of GC patients according to combinations of IL-34, M-CSF and CD68^+^-TAMs (Fig. 3). Patients with high IL-34 plus high CD68^+^-TAMs had the longest survival of GC patients, while those with low IL-34 plus low CD68^+^-TAMs had the lowest survival. However, there was no significant difference in survival for the combination of IL-34 and M-CSF, or CD68^+^-TAMs and M-CSF.

Furthermore, to determine whether IL-34 was an independent prognostic marker for GC, we performed univariate and multivariate Cox regression analysis, including IL-34, age, gender, tumour differentiation, lymph node invasion, tumour size, the depth of tumour invasion and TNM stage. The effect of IL-34 on patient survival in GC was determined. Univariate analysis (Table 2) revealed that the expression of IL-34, advanced TNM stage, lymph node metastasis, the depth of tumour invasion and tumour diameter were correlated with the prognosis of GC patients. In multivariate analysis (Table 2), only advanced TNM stage remained a significant independent prognostic factor for the survival of patients.

**Table 2:** Univariate and multivariate analysis of IL-34 and clinicopathological factors affecting survival of patients with GC

Variables	Univariate analysis		Multivariate analysis	
	HR (95% CI)	*p*-value	HR (95% CI)	*p*-value
IL-34 (low/high)	2.205 (1.072–4.537)	***0.032***	1.457 (0.639–3.325)	0.371
Gender				
Female/male	0.886 (0.443–1.775)	0.733		
Age (≤ 60/> 60)	1.058 (0.573–1.955)	0.857		
Diameter (< 5/≥ 5, cm)	0.363 (0.189–0.698)	***0.002***	0.495 (0.217–1.129)	0.095
Lymph node metastasis				
No/yes	0.267 (0.122–0.582)	***0.001***	0.772 (0.274–2.173)	0.624
Tumour differentiation				
Low (reference)	1	0.545		
High	0.725 (0.218–2.411)	0.600		
Moderate	0.693 (0.349–1.375)	0.294		
Invasion depth				
T4	1	0.142	1	0.955
T1	0.000 (0.000–)	0.977	0.900 (0.000–)	1.000
T2	0.261 (0.084–0.808)	***0.020***	1.528 (0.326–7.165)	0.591
T3	0.750 (0.380–1.478)	0.405	1.049 (0.463–2.378)	0.909
TNM				
IV (reference)	1	***0.000***	1	***0.001***
I	0.000 (0.000–)	0.966	0.000 (0.000–)	0.969
II	0.085 (0.028–0.260)	***0.000***	0.075 (0.021–0.270)	***0.000***
III	0.195 (0.068–0.559)	***0.002***	0.154 (0.047–0.503)	***0.002***

Bold italic values indicate significance (*p* < 0.05)

### Further analysis of correlation of M-CSF and CD68^+^-TAMs with overall survival in subgroups of GC patients

**Fig. 4:**
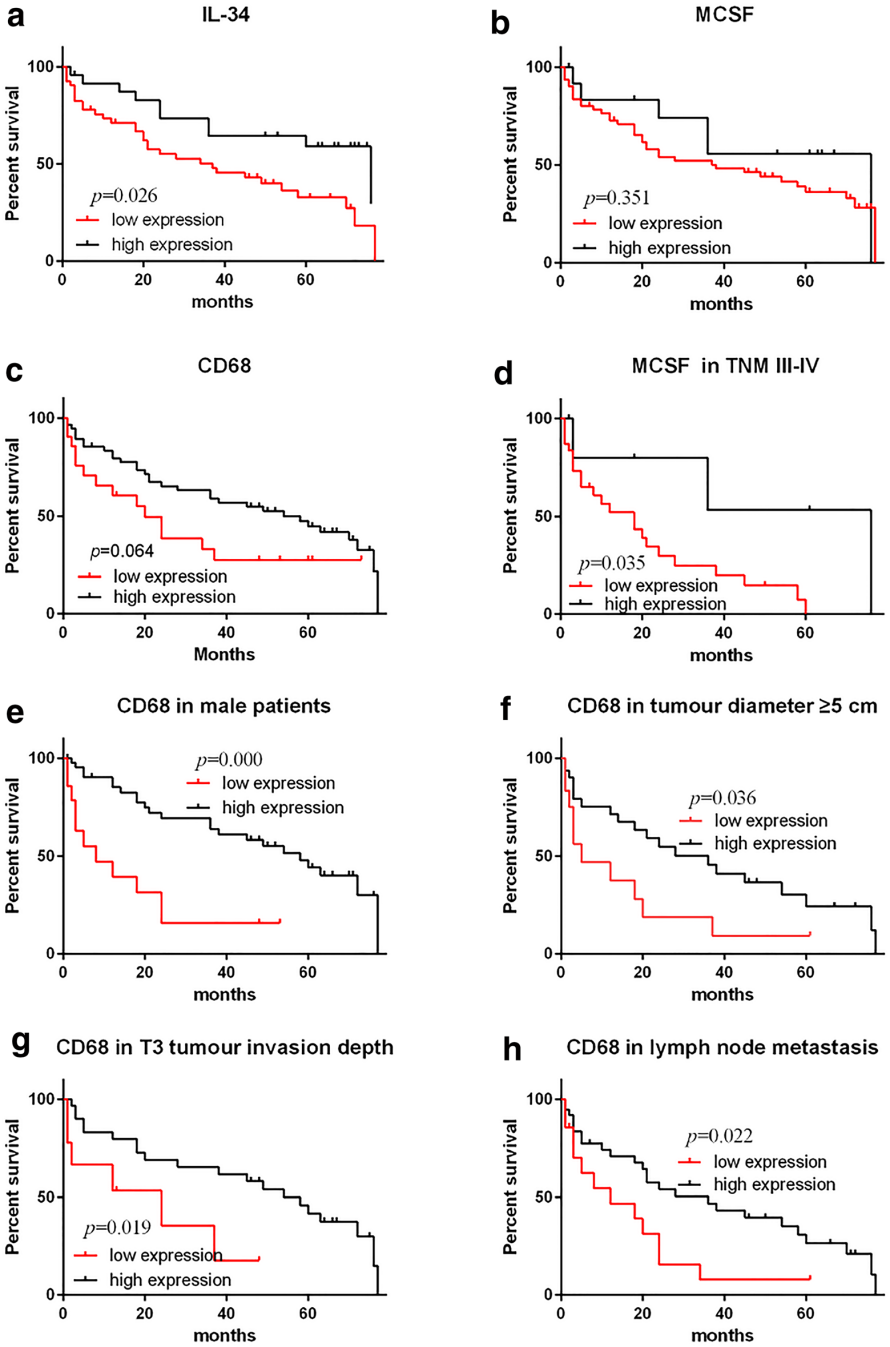
Survival analysis of IL-34, MCSF and CD68^+^ TAMs for prognosis of GC. Kaplan–Meier survival analysis of GC patients: decreased IL-34 expression correlates with a poor survival for GC patients. Decreased MCSF expression correlates with poor survival of GC patients in TNM III-IV stage. Increased CD68^+^ TAMs expression correlates with good survival of GC patients in male, Diameter ≥ 5 cm, lymph node metastasis and T3 stage

Kaplan–Meier analysis was also applied to further compare overall survival according to M-CSF and CD68^+^-TAMs in different subgroups of GC. As results show in Fig. 4, M-CSF correlated significantly with the survival of patients in the TNM III-IV tumour stage. CD68^+^-TAMs correlated with survival significantly in male GC patients, larger tumour size (diameter ≥ 5 cm), lymph node metastasis and tumour invasion depth T3. There was no significance in other subgroups between survival and M-CSF and CD68^+^-TAMs (Additional file 3: Figure S3, Additional file 4: Figure S4, Additional file 5: Figure S5, Additional file 6: Figure S6).

Furthermore, to examine whether M-CSF and CD68^+^-TAMs were independent prognostic markers for subgroups in GC, we again performed univariate and multivariate Cox regression analysis, including M-CSF and CD68^+^-TAMs, age, gender, tumour differentiation, lymph node invasion, tumour size, the depth of tumour invasion and TNM stage to study the effects of M-CSF and CD68^+^-TAMs on patient survival in GC subgroups.

Using univariate analysis a correlation was observed between survival of GC patients and CD68^+^ TAMs, TNM stage or lymph node metastasis in the T3 stage subgroup of GC patients, respectively (Table 3). Using multivariate analysis, it was demonstrated that CD68^+^ TAMs and TNM stage remained as significant independent prognostic factors for survival of GC patients within these subgroups.

**Table 3:** Univariate and multivariate analysis of CD68^+^ TAMs and clinicopathological factors affecting survival of patients in T3 stage of GC

Variables	Univariate analysis		Multivariate analysis	
	HR (95% CI)	*p*-value	HR (95% CI)	*p*-value
CD68 (low/high)	3.080 (1.135–8.360)	***0.027***	5.471 (1.825–16.397	***0.002***
Gender				
Female/male	0.638 (0.216–1.886)	0.416		
Age (≤ 60/> 60)	1.052 (0.479–2.310)	0.900		
Diameter(< 5/≥ 5, cm)	0.464 (0.196–1.100)	0.081		
Lymph node metastasis				
No/yes	0.407 (0.167–0.993)	***0.048***	0.363 (0.123–1.075)	0.067
Tumour differentiation				
Low (reference)	1	0.436		
High	0.269 (0.035–2.059)	0.206		
Moderate	0.825 (0.348–1.955)	0.663		
TNM				
IV (reference)	1	***0.019***	1	***0.007***
II	0.140 (0.029–0.683)	***0.015***	0.061 (0.011–0.351)	***0.002***
III	0.337 (0.070–1.619)	***0.174***	0.101 (0.016–0.634)	***0.014***

Bold italic values indicate significance (*p* < 0.05)

Using univariate analysis, it was demonstrated that there is a correlation between survival of GC patients and CD68^+^ TAMs, tumour diameter, advanced TNM stage and lymph node metastasis in the male GC patients’ subgroup (Table 4). Multivariate analysis demonstrated that CD68^+^ TAMs, tumour diameter ≥ 5 cm and advanced TNM stage remained as significant independent prognostic factors of survival of male GC patients.

**Table 4:** Univariate and multivariate analysis of CD68^+^ TAMs and clinicopathological factors affecting survival of ***male*** patients of GC

Variables	Univariate analysis		Multivariate analysis	
	HR (95% CI)	*p*-value	HR (95% CI)	*p*-value
CD68 (low/high)	3.843 (1.773–8.329)	***0.001***	3.905 (1.599–9.538)	***0.003***
Age (≤ 60/> 60)	0.876 (0.441–1.740)	0.706		
Diameter(< 5 vs ≥ 5, cm)	0.308 (0.150–0.636)	***0.001***	0.393 (0.172–0.901)	***0.027***
Lymph node metastasis				
No/yes	0.300 (0.133–0.677)	***0.004***	0.847 (0.328–2.186)	0.731
Tumour differentiation				
Low (reference)	1	0.737		
High	0.633 (0.186–2.153)	0.464		
Moderate	0.848 (0.402–1.790)	0.666		
Invasion depth				
T4	1	0.366		
T1	0.000 (0.000–)	0.975		
T2	0.300 (0.080–1.129)	0.075		
T3	0.740 (0.332–1.652)	0.462		
TNM				
IV (reference)	1	***0.002***	1	***0.040***
I	0.000 (0.000–)	0.978	0.000 (0.000–)	0.977
II	0.104 (0.030–0.356)	***0.000***	0.137 (0.035–0.529)	***0.004***
III	0.299 (0.094–0.948)	0.299	0.232 (0.066–0.816)	***0.023***

Bold italic values indicate significance (*p* < 0.05)

Using univariate analysis for survival in the subgroup of tumour diameter ≥ 5 cm (Table 5) and lymph node metastasis subgroup (Table 6), CD68^+^-TAMs and TNM stage were correlated with the prognosis of GC patients within these two subgroups. However, only TNM stage remained a significant independent prognostic factor of survival of GC patients in multivariate analysis in both subgroups. In the TNM III–IV subgroup of GC patients there was no significant outcome in M-CSF using univariate analysis (Additional file 7: Table S1).

**Table 5:** Univariate and multivariate analysis of CD68^+^ TAMs and clinicopathological factors affecting survival of GC in subtype of ***diameter *≥ *5*** ***cm***

Variables	Univariate analysis		Multivariate analysis	
	HR (95% CI)	*p*-value	HR (95% CI)	*p*-value
CD68 (low/high)	2.222 (1.014–4.872)	***0.046***	1.781 (0.776–4.087)	0.173
Gender				
Female/male	0.778 (0.349–1.733)	0.539		
Age (≤ 60/> 60)	0.924 (0.442–1.928)	0.832		
Lymph node metastasis				
No/yes	0.820 (0.311–2.165)	0.689		
Tumour differentiation				
Low (reference)	1	0.533		
High	1.100 (0.144–8.377)	0.927		
Moderate	0.636 (0.284–1.426)	0.272		
Invasion depth				
T4	1	0.548		
T2	0.610 (0.131–2.836)	0.529		
T3	0.645 (0.286–1.453)	0.290		
TNM				
IV (reference)	1	***0.007***	1	***0.025***
II	0.095 (0.022–0.412)	***0.002***	0.125 (0.027–0.568)	***0.007***
III	0.157 (0.040–0.616)	***0.008***	0.184 (0.047–0.724)	***0.015***

Bold italic values indicate significance (*p* < 0.05)

**Table 6:** Univariate and multivariate analysis of clinicopathological factors affecting survival of GC in subtype of ***lymph node metastasis***

Variables	Univariate analysis		Multivariate analysis	
	HR (95% CI)	*p*-value	HR (95% CI)	*p*-value
CD68 (low/high)	2.220 (1.083–4.553)	***0.029***	1.559 (0.706–3.443)	0.272
Gender				
Female/male	0.968 (0.472–1.988)	0.930		
Age (≤ 60/> 60)	0.957 (0.497–1.843)	0.896		
Diameter (< 5/≥ 5 cm)	0.773 (0.384–1.557)	0.472		
Tumour differentiation				
Low (reference)	1	0.360		
High	0.772 (0.230–2.598)	0.676		
Moderate	0.570 (0.263–1.237)	0.155		
Invasion depth				
T4		0.529		
T2	0.538 (0.174–1.660)	0.281		
T3	0.774 (0.378–1.584)	0.483		
TNM				
IV (reference)	1	***0.001***	1	***0.011***
II	0.083 (0.022–0.314)	***0.000***	0.113 (0.027–0.476)	***0.003***
III	0.152 (0.046–0.498)	***0.002***	0.188 (0.055–0.647)	***0.008***

Bold italic values indicate significance (*p* < 0.05)

## Discussion

We have demonstrated that decreased IL-34 in GC is inversely correlated with tumour differentiation, age and female gender of GC patients, which is consistent with others showing that younger, particularly female GC patients had more severe malignancy than older, male patients [23, 24]. Decreased IL-34 was closely related to the poor survival rate of GC patients, but not as an independent prognostic factor. Liu et al. demonstrate that > 60% of GC patients had low M-CSF, using positive expression rate only [25]. This is in line with our current study revealed that M-CSF was decreased in GC compared to the control. However, there was no correlation between M-CSF and clinical–pathological parameters, as well as, prognosis of GC in the current study. Interestingly, in the TNM III–IV subtype of GC, decreased M-CSF was inversely correlated with prognosis of GC, but M-CSF still cannot be considered an independent prognostic factor.

IL-34 induces differentiation of leukaemia cells into monocyte-like, macrophage-like cells and mature macrophages through the JAK/STAT and PI3K/Akt signalling pathways [26, 27], suggesting that IL-34 enhances differentiation of other cancers, and supporting our current finding that IL-34 was correlated with the differentiation of GC. Our data may provide an explanation for the possible role of IL-34 in the development of GC, i.e. IL-34 also regulates GC differentiation, which would have potential clinical relevance regarding IL-34 as a therapeutic target for malignancy.

IL-34 and M-CSF can induce macrophage polarization, mainly into the M2 phenotype, which subsequently leads to M2 macrophage mediated immunosuppression [28], promoting tumour progression and metastasis [26]. However, a controversial report showed no significant correlation between the serum level of M-CSF and the stage and prognosis in GC patients [29]. Anti-M-CSF antibody doesn’t induce cytotoxic effects on breast cancer in vitro, which may be either due to a differential effect in these different cancer models and/or variance between in vivo and in vitro [30]. Additionally, IL-34- and MCSF-induced macrophages can switch memory T cells into Th17 cells [18] to support anti-tumour immunity in established ovarian cancers [31].

Macrophage mediated host cellular immunity is important in tumour oncogenesis [32]. Monocytes differentially polarize into M1 and M2 macrophages [33], but in TAMs M1 and M2 polarization is rarely observed [34]. CD68 is frequently used as a marker of infiltrated TAMs, regardless of their polarization state in many studies. Our data show there was no correlation between CD68^+^-TAMs and any clinicopathological parameters, as well as, prognosis of GC, except for increased number of CD68^+^-TAMs in GC. We found there was better prognosis in GC patients with high CD68^+^-TAMs in males, with GC size ≥ 5 cm, lymph node metastasis and T3 subtypes. This is consistent with findings in colon and colorectal cancers [35, 36], but not in other cancer [37, 38].

TAMs are mixed phenotype, expressing M1 or M2 markers [39], and may be influenced by different microenvironments in different regions and/or in different individuals. The functions of TAMs can be modified by cancer and cancer cell secretions, which in turn could affect tumour growth and differentiation [40]. However, our current observation invites speculation that the increased infiltrating CD68^+^-TAMs may be M1 dominant, contributing to anti-tumour activity, which will be determined in our future experiments. The possible relationship between M1 vs M2 and IL-34 has been recently reviewed [41].

A significant correlation was observed between the combination of IL-34/CD68^+^-TAMs and the prognosis of GC. Tumours with high IL-34 plus high CD68^+^-TAMs had the best prognosis, tumours with high or low IL-34 plus low or high CD68^+^-TAMs had mid-level prognosis and tumours with low IL-34 plus low CD68^+^-TAMs had the worst prognosis in GC patients. Our results verify a significant correlation between the combination of IL-34 and CD68^+^-TAMs and prognosis of GC patients. The precise underlying mechanism of IL-34/M-CSF/M-CSFR axis in tumorigenesis, particular in GC, will be determined in future work. Finally, our data may also provide useful information in personalised decision making for precision medicine, which may substantially reduce adverse effects of chemotherapy, and improve the outcome.

At this stage, our data demonstrate that IL-34 and its related markers M-CSF and TAM correlated well with prognosis of gastric cancer patients, particularly in the male gastric cancer patients. Our observation suggests IL-34 might be a potential biomarker for predicting the prognosis of gastric cancer patients. However, there is still a long way to go before IL-34 can be used for this purpose. This practical issue is currently being investigated using IL-34 transgenic and IL-34 gene knockout mice, as well as further investigation ex vivo in human tissues. Clinical application is also being examined in gastric biopsy samples.

Although our data suggest that IL-34 and CD68^+^ TAM might be useful biomarkers in gastric cancer, other factors, for example, Epstein–Barr virus infection, are well known to be linked with gastric cancer [42]. Such linkage will be determined in our future studies.

In our study we have compared cancer tissue with adjacent normal gastric mucosa from the same patients as a control. Ideally, we should use normal gastric tissue from non-cancer patients, however, due to ethics issues we are not able to obtain normal gastric tissue from non-cancer patients for our research purposes. Perhaps in the future we may collect normal gastric tissue from organ donors, e.g. heart, lung, kidney donors. Our current observation is based on immunohistochemistry exclusively. However, it remains to be clarified whether mRNA for IL-34, M-CSF and macrophages markers are also expressed in the same way, which will be determined in our future experiments. A tissue array methodology was used in the current study for immunohistochemical staining. Thus, due to limited size of the tumour sample within the array, we are not able to demonstrate the expression of CD68^+^-TAMs and the loss of IL-34/M-CSF at the interface of tumour invasion region in the existing immunohistochemically stained slides. We also are unable to undertake more fresh immunostaining at this stage, due to COVID-19 limitations for undertaking any wet laboratory experiments in the University of Sydney. This excellent suggestion will be performed in our future experiments. Finally, as stated above, there is overlapping functions between IL-34 and M-CSF, which may explain why IL-34 is not an independent prognostic factor in GC.

## Conclusion

Reduced IL-34 was associated with poor differentiation, poor survival rate, relatively young patients and female GC patients. IL-34 may serve as a predictive biomarker, but not as an independent, prognostic factor in GC. M-CSF inversely correlated with survival of GC in TNM III–IV subtype, but was also not an independent prognostic factor. Increased CD68^+^-TAMs were a good prognostic factor of GC in male, tumour diameter ≥ 5 cm, lymph node metastasis and T3 subgroups. CD68^+^-TAMs could therefore be used as an independent prognostic factor in male T3 stage GC. Furthermore, the combination of IL-34 and CD68^+^-TAMs might serve as a useful prognostic marker in GC. These results collectively support the potency of IL-34, M-CSF, CD68^+^-TAMs and the combination of IL-34 and CD68^+^-TAMs as novel biological markers for GC, thus may provide new insight for both diagnosis and cellular therapy of GC.

## Methods

### Patients and samples

GC tissue and adjacent histologically normal gastric tissue (control) was obtained from 180 GC patients undergoing gastrectomy without prior chemotherapy in Xuzhou Medical University, China (2008–2010). These GC patients were 140 males and 40 females (aged 23–85 years) (Table 1) with complete clinical information. Among them, 77 had follow-up until their death or until their most recent contact (May, 2015). At the time of the most recent contact, 14 of 77 were still alive, whereas 42 were dead and the other 21 were lost contacts during the following-up. Among the 14 surviving patients, the longest survival period was 76 months. The tissues within the pathology blocks were obtained from the patents at surgery. The consent for surgery included consent for the tissues to be used for diagnostic and research purpose in an unidentified manner. A written explanation of the surgical procedures and the potential research use of the tissues was provided to the patient prior to surgery. All of the patients were adults who were older than 16 years. This study was approved by the Human Ethical Committee, Xuzhou Medical University (xyfylw2012002).

### Immunohistochemistry

Sections (5 µm) from tissue microarray blocks were labelled with three antibodies, as described previously [43]. The antibodies were: rabbit anti-IL-34 polyclonal antibody (bs-18170R, Beijing Biosynthesis Biotechnology, China), rabbit anti-M-CSF (Abcam, Cambridge, UK) and mouse monoclonal anti-CD68 (Dako, Copenhagen, Denmark). HRP-conjugated secondary antibody (Beijing Sequoia Jinqiao Biological Technology) was used. The specific target(s) was visualized with 3, 3′-diaminobenzidine (DAB) detection kit and counterstained with hematoxylin. IL-34, M-CSF and CD68 production was quantified.

Briefly, all the images were taken by an Olympus BX51 microscope with fixed exposure time and light sources to avoid any additional unwanted errors. The quantitative analysis was conducted using ImagePro Plus 7.1 software (Media Cybernetics, Silver Spring, MD) as described by Liu et al. [43–45]. The positive staining threshold was defined by an independent pathologist in a double-blind fashion. The defined threshold was applied to analyse all of the images, using a pre-programmed macro in ImagePro Plus 7.1 software to obtain the objective positive value (pixels) of the staining. The positive pixels were expressed as image units. The mean of these values represents the amount of staining per treatment group used for subsequent statistical comparison, as described below.

### Statistical analysis

The SPSS 16.0 statistical software package was used for the statistical analysis. Comparison between two groups was performed via Mann–Whitney U test, as described [44, 45]. Comparisons among multi-groups were performed via Kruskal–Wallis test. Low and high cutoff values for cytokine expression was defined by ROC curve analysis [46]. Survival curves were plotted by the Kaplan–Meier method and compared by the log-rank test. Cox’s proportional hazards model was used to identify the prognostic factors that influenced survival. *p *< 0.05 was considered statistically significant.

## Supplementary information

**Additional file 1: Figure S1.** Correlation of IL-34, MCSF and CD68^+^ TAMs expression with clinicopathological parameters of tumour size, lymph node metastasis, tumour invasion depth and TNM subtypes of GC. IL-34, MCSF and CD68^+^ TAMs all have no correlations with any clinicopathological parameters of tumour size, lymph node metastasis, tumour invasion depth and TNM subtypes of GC.

**Additional file 2: Figure S2.** Correlation of MCSF and CD68^+^ TAMs with clinicopathological parameters of age, gender and differentiation subtypes of GC. MCSF and CD68^+^ TAMs both have no correlations with any clinicopathological parameters of age, gender and differentiation subtypes of GC.

**Additional file 3: Figure S3.** Survival analysis of MCSF for prognosis of subtypes of GC patients. Kaplan-Meier survival analysis of GC patients: there were no correlations of MCSF with survival of gender, age, diameter and lymph node metastasis subtypes of GC patients.

**Additional file 4: Figure S4.** Survival analysis of MCSF for prognosis of subtypes of GC patients. Kaplan-Meier survival analysis of MCSF for prognosis of GC in differentiation, tumour invasion depth and TNM stage subtypes.

**Additional file 5: Figure S5.** Survival analysis of CD68^+^ TAMs for prognosis of subtypes of GC patients. Kaplan-Meier survival analysis of CD68^+^ TAMs for prognosis of GC in female, tumour diameter < 5 cm, no lymph node metastasis and T4 stage subtypes.

**Additional file 6: Figure S6.** Survival analysis of CD68^+^ TAMs for prognosis of subtypes of GC patients. Kaplan-Meier survival analysis of CD68^+^ TAMs for prognosis of GC in age, tumour differentiation and TNM stage subtypes.

**Additional file 7: Table S1.** Univariate analysis of MCSF and clinicopathological factors affecting survival of patients with GC in TNM III–IV.

## Data Availability

Yes.

## Abbreviations

CRC: Gastric cancer
IL: Interleukin
TAM: Tumour associate macrophages
M-CSF: Macrophage colony stimulating factor
AUC: Area under the curve
ROC: Receiver operating characteristic
TNM: Tumour, node and metastasis
NFkb: Nuclear factor kappa B
MAPKs: Mitogen-activated protein kinase
DCs: Dendritic cells
