# Supplementary material for: Inverse correlation between Interleukin-34 and gastric cancer, a potential biomarker for prognosis
Source: Cell Biosci. 2020 Aug 4;10:94. doi: 10.1186/s13578-020-00454-8 (PMC7399616; doi:10.1186/s13578-020-00454-8)

**Figure S1** Correlation of IL-34, MCSF and CD68^+^ TAMs with clinicopathological parameters of tumour size, lymph node metastasis, tumour invasion depth and TNM subtypes of GC


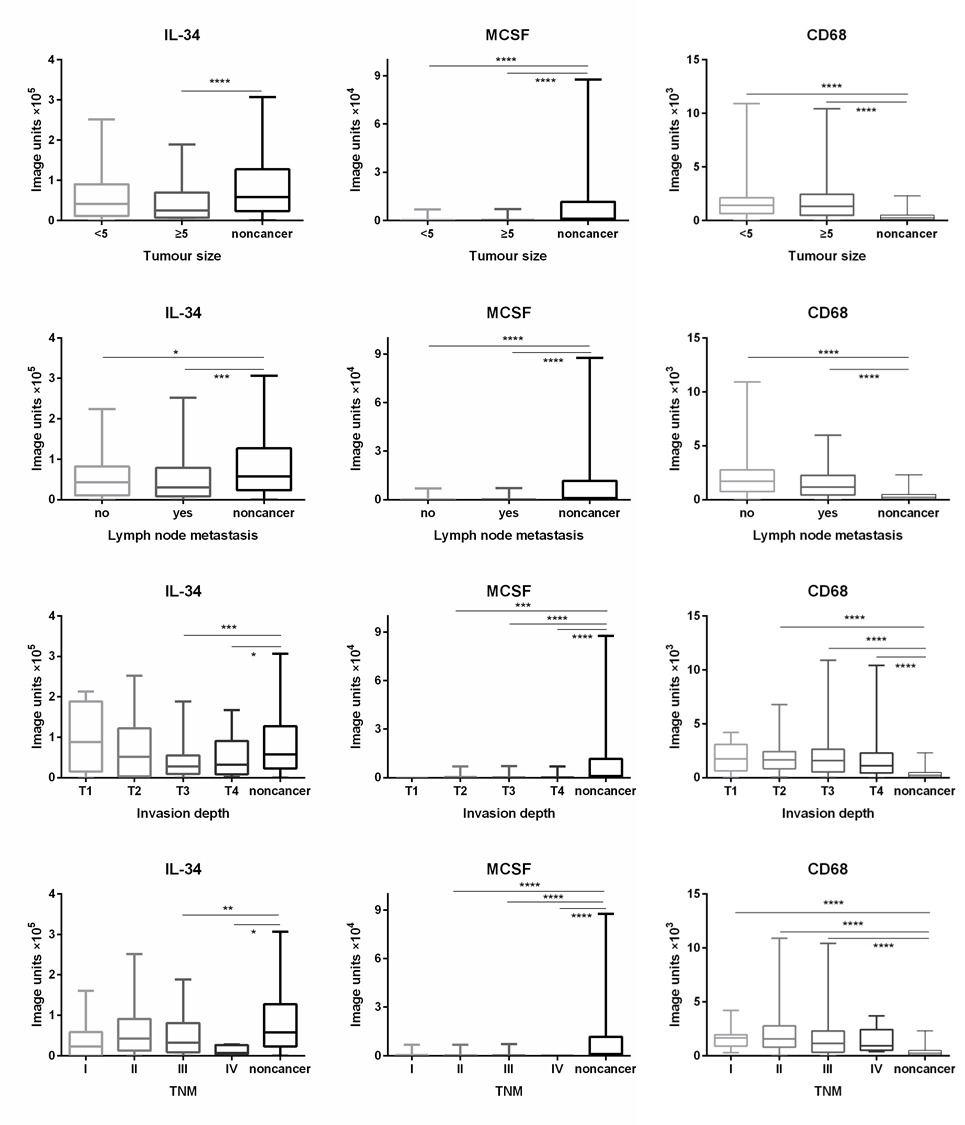

Supplement: Supplementary file 1 — Additional file 1: Figure S1. Correlation of IL-34, MCSF and CD68+ TAMs expression with clinicopathological parameters of tumour size, lymph node metastasis, tumour invasion depth and TNM subtypes of GC. IL-34, MCSF and CD68+ TAMs all have no correlations with any clinicopathological parameters of tumour size, lymph node metastasis, tumour invasion depth and TNM subtypes of GC. [file 13578_2020_454_MOESM1_ESM.docx]
