# Supplementary material for: Inverse correlation between Interleukin-34 and gastric cancer, a potential biomarker for prognosis
Source: Cell Biosci. 2020 Aug 4;10:94. doi: 10.1186/s13578-020-00454-8 (PMC7399616; doi:10.1186/s13578-020-00454-8)

**Figure S2** Correlation of MCSF and CD68^+^ TAMs with clinicopathological parameters of age, gender and differentiation subtypes of GC


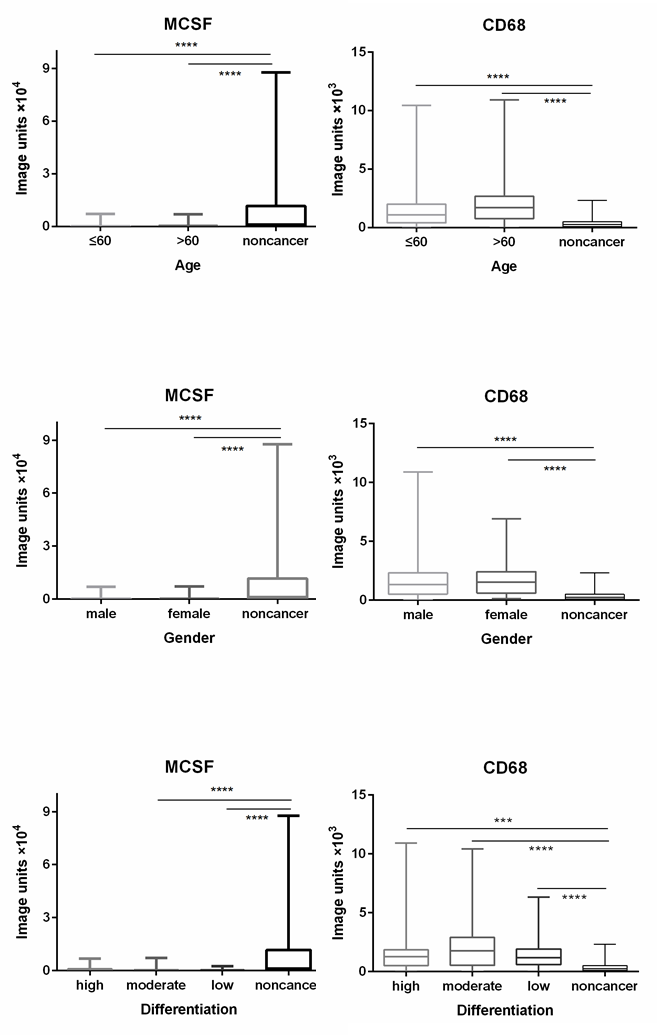

Supplement: Supplementary file 2 — Additional file 2: Figure S2. Correlation of MCSF and CD68+ TAMs with clinicopathological parameters of age, gender and differentiation subtypes of GC. MCSF and CD68+ TAMs both have no correlations with any clinicopathological parameters of age, gender and differentiation subtypes of GC. [file 13578_2020_454_MOESM2_ESM.dotx]
