# Supplementary material for: Inverse correlation between Interleukin-34 and gastric cancer, a potential biomarker for prognosis
Source: Cell Biosci. 2020 Aug 4;10:94. doi: 10.1186/s13578-020-00454-8 (PMC7399616; doi:10.1186/s13578-020-00454-8)
Supplement: Supplementary file 3 — Additional file 3: Figure S3. Survival analysis of MCSF for prognosis of subtypes of GC patients. Kaplan-Meier survival analysis of GC patients: there were no correlations of MCSF with survival of gender, age, diameter and lymph node metastasis subtypes of GC patients. [file 13578_2020_454_MOESM3_ESM.docx]

**Figure S3** Survival analysis of MCSF for prognosis of subtypes of GC patients

**

**
