# Supplementary material for: Inverse correlation between Interleukin-34 and gastric cancer, a potential biomarker for prognosis
Source: Cell Biosci. 2020 Aug 4;10:94. doi: 10.1186/s13578-020-00454-8 (PMC7399616; doi:10.1186/s13578-020-00454-8)
Supplement: Supplementary file 4 — Additional file 4: Figure S4. Survival analysis of MCSF for prognosis of subtypes of GC patients. Kaplan-Meier survival analysis of MCSF for prognosis of GC in differentiation, tumour invasion depth and TNM stage subtypes. [file 13578_2020_454_MOESM4_ESM.docx]

**Figure S4** Survival analysis of MCSF for prognosis of subtypes of GC patients

**

**
