# Supplementary figures and images for: Inverse correlation between Interleukin-34 and gastric cancer, a potential biomarker for prognosis
Source: Cell Biosci. 2020 Aug 4;10:94. doi: 10.1186/s13578-020-00454-8 (PMC7399616; doi:10.1186/s13578-020-00454-8)

**Figure S5** Survival analysis of CD68+ TAMs for prognosis of subtypes of GC patients

**
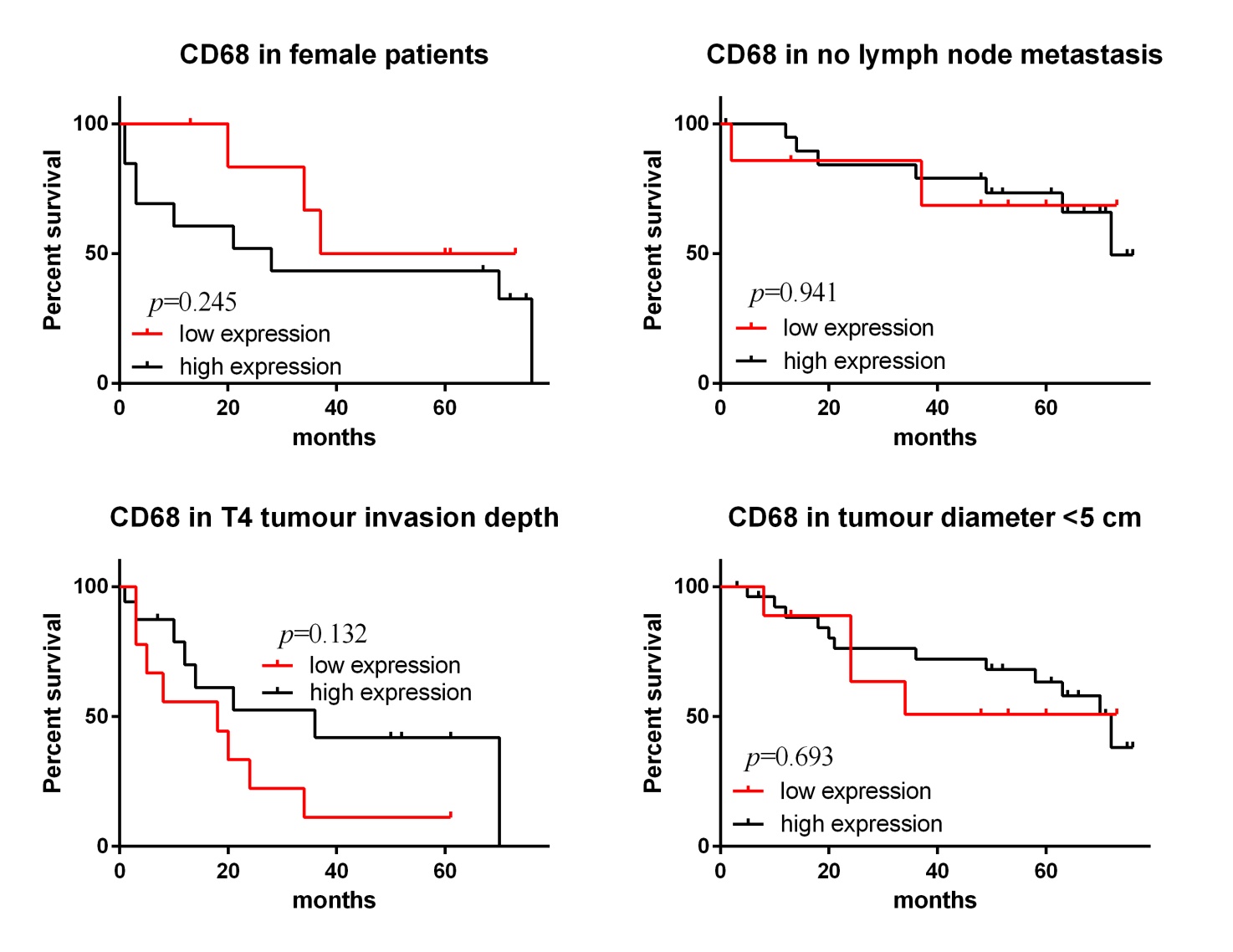
**

Supplement: Supplementary file 5 — Additional file 5: Figure S5. Survival analysis of CD68+ TAMs for prognosis of subtypes of GC patients. Kaplan-Meier survival analysis of CD68+ TAMs for prognosis of GC in female, tumour diameter < 5 cm, no lymph node metastasis and T4 stage subtypes. [file 13578_2020_454_MOESM5_ESM.docx]
