# Supplementary material for: Inverse correlation between Interleukin-34 and gastric cancer, a potential biomarker for prognosis
Source: Cell Biosci. 2020 Aug 4;10:94. doi: 10.1186/s13578-020-00454-8 (PMC7399616; doi:10.1186/s13578-020-00454-8)
Supplement: Supplementary file 6 — Additional file 6: Figure S6. Survival analysis of CD68+ TAMs for prognosis of subtypes of GC patients. Kaplan-Meier survival analysis of CD68+ TAMs for prognosis of GC in age, tumour differentiation and TNM stage subtypes. [file 13578_2020_454_MOESM6_ESM.docx]

**Figure S6** Survival analysis of CD68+ TAMs for prognosis of subtypes of GC **

**

patients
