# Supplementary material for: Inverse correlation between Interleukin-34 and gastric cancer, a potential biomarker for prognosis
Source: Cell Biosci. 2020 Aug 4;10:94. doi: 10.1186/s13578-020-00454-8 (PMC7399616; doi:10.1186/s13578-020-00454-8)
Supplement: Supplementary file 7 — Additional file 7: Table S1. Univariate analysis of MCSF and clinicopathological factors affecting survival of patients with GC in TNM III–IV. [file 13578_2020_454_MOESM7_ESM.docx]

**Table S1** Univariate analysis of MCSF and clinicopathological factors affecting survival of patients with GC in TNM III- IV

| Variables | Univariate analysis | |
| --- | --- | --- |
|  | HR (95%CI) | *p*-value |
| **MCSF (low/high)** | 3.980(0.923-17.161) | 0.064 |
| **Gender** |  |  |
| Female/male | 0.671(0.274-1.643) | 0.383 |
| **Age (≤60/>60)** | 1.314(0.593-2.915) | 0.501 |
| **Diameter(<5/≥5, cm)** | 0.749(0.313-1.793) | 0.516 |
| **Lymph node metastasis** | |  |
| No/yes | 1.186(0.275-5.121) | 0.819 |
| **Tumour differentiation** | |  |
| Low (reference) | 1 | 0.861 |
| High | 0.598(0.078-4.594) | 0.621 |
| Moderate | 0.876(0.362-2.117) | 0.769 |
| **Invasion depth (**T3/T4**)** | 0.991(0.449-2.187) | 0.982 |
